# Prednisone for the prevention of tuberculosis-associated IRIS (randomized controlled trial): Impact on the health-related quality of life

**DOI:** 10.3389/fpsyg.2022.983028

**Published:** 2022-10-05

**Authors:** Edwin Wouters, Cari Stek, Alison Swartz, Jozefien Buyze, Charlotte Schutz, Friedrich Thienemann, Robert J. Wilkinson, Graeme Meintjes, Lutgarde Lynen, Christiana Nöstlinger

**Affiliations:** ^1^Centre for Population, Family & Health, University of Antwerp, Antwerp, Belgium; ^2^Centre for Health Systems Research & Development, University of the Free State, Bloemfontein, South Africa; ^3^Department of Clinical Sciences, Institute of Tropical Medicine, Antwerp, Belgium; ^4^Wellcome Centre for Infectious Diseases Research in Africa, Institute of Infectious Disease and Molecular Medicine, University of Cape Town, Cape Town, South Africa; ^5^Division of Social and Behavioural Sciences, School of Public Health and Family Medicine, University of Cape Town, Cape Town, South Africa; ^6^Department of Medicine, University of Cape Town, Cape Town, South Africa; ^7^Department of Medicine, Imperial College London, London, United Kingdom; ^8^The Francis Crick Institute, London, United Kingdom

**Keywords:** tuberculosis, TB-IRIS, prednisone, quality of life, South Africa

## Abstract

**Background:**

Tuberculosis-associated immune reconstitution inflammatory syndrome (TB-IRIS) is an important complication in patients with HIV-associated tuberculosis (TB) starting antiretroviral treatment (ART) in sub-Saharan Africa. The PredART-trial recently showed that prophylactic prednisone reduces the incidence of paradoxical TB-IRIS by 30% in a population at high risk. This paper reports the impact of the intervention on health-related quality of life (HRQoL), a secondary endpoint of the trial, measured by an amended version of the PROQOL-HIV instrument—the instrument’s validity and reliability is also assessed.

**Methods:**

A total of 240 adult participants (antiretroviral treatment (ART)-naïve, TB-HIV co-infected with CD4 count ≤100 cells/μL) were recruited and randomized (1:1) to (1) a prednisone arm or (2) a placebo arm. In this sub-study of the PredART-trial we evaluated (1) the performance of an HIV-specific HR-QoL instrument amended for TB-IRIS, i.e., the PROQOL-HIV/TB in patients with HIV-associated TB starting ART (reliability, internal and external construct validity and invariance across time) and (2) the impact of prednisone on self-reported HR-QoL in this population through mixed models.

**Results:**

The PROQOL-HIV/TB scale displayed acceptable internal reliability and good internal and external validity. This instrument, including the factor structure with the eight sub-dimensions, can thus be applied for measuring HR-QoL among HIV-TB patients at high risk for TB-IRIS. Prophylactic prednisone was statistically significantly associated only with the ‘Physical Health and Symptoms’-subscale: a four-week course of prednisone resulted in an earlier improvement in the physical dimension of HR-QoL compared to placebo.

**Conclusion:**

We demonstrated that the PROQOL-HIV/TB scale adequately measures different aspects of self-reported HR-QoL in HIV-TB patients. Although more research is needed to understand how other domains related to HR-QoL can be improved, targeting patients at high risk for developing TB-IRIS with a four-week course of prednisone has a beneficial effect on the physical aspects of patient-reported quality of life.

## Introduction

HIV/AIDS and tuberculosis (TB) have merged into a deadly co-epidemic in South Africa. In absolute numbers, the country has the highest number of people living with HIV [7.8 million (or 13,151 per 100,000) in 2020; Ndlovu et al., 2021]. In addition, it has one of the most severe TB epidemics in the world, with the second highest incidence of TB (552 per 100,000 in 2020; Ndlovu et al., 2021). These epidemics are intricately intertwined (Wouters et al., 2020): approximately 60% of patients with TB are co-infected with HIV (WHO, 2018).

Starting patients with HIV-associated TB on antiretroviral therapy (ART) is essential and can be live-saving (Cohen and Meintjes, 2010). Accordingly, the South African government’s 2016 Universal Test and Treat program stipulates to start TB treatment in co-infected patients and follow with ART as soon as possible and within 8 weeks [SA-NDoH circular, 2016, New HIV Guideline: Universal Test and Treat (UTT)]. In 2020, 89% of new and relapse TB patients (with a known HIV status) who were HIV positive were (started) on ART (WHO, 2021).

However, paradoxical tuberculosis-associated immune reconstitution inflammatory syndrome (TB-IRIS) complicates treatment in 18% (95%CI 16–21%) of patients, with percentages up to 50% in high risk groups (i.e., low CD4 counts and a short interval between starting anti-tuberculosis treatment and ART). TB-IRIS is an immunopathological reaction, resulting in new, recurrent, or worsening signs or symptoms of TB. It usually occurs within the first few weeks after starting ART (Namale et al., 2015; Sereti et al., 2019). This entails risks as TB-IRIS has been associated with lower ART adherence rates (Nachega et al., 2012) and higher risk of hospitalization (Namale et al., 2015).

The PredART trial recently showed that prophylactic prednisone reduces the incidence of paradoxical TB-IRIS within 12 weeks of starting ART (primary efficacy endpoint of the trial) by 30% in a population at high risk (i.e., HIV-infected, ART-naïve adult patients diagnosed with active TB who have a CD4 < 100 cells/μL and who start ART within 30 days of starting TB treatment) (Meintjes et al., 2018). One of the trial’s secondary endpoints was the effect of prednisone on health-related quality of life (HRQoL). Previous studies have indicated that patients display lower levels of adherence to ART when they experience deteriorations in their quality of life (measured by the HIV/AIDS-Targeted Quality of Life instrument and the Short Form-12; Mannheimer et al., 2005; Holmes et al., 2007). It is therefore relevant to see whether the impact of prophylactic prednisone on the incidence of TB-IRIS is also reflected in improvements in HRQoL as the absence of these improvements could potentially negatively impact adherence to Prednisone and curtail its positive impact.

Studies investigating HRQoL in patients with HIV-associated TB, however, are limited and either use HIV-specific or generic HRQoL measurement instruments (Deribew et al., 2013; Dowdy et al., 2013; Louw et al., 2016; Mthiyane et al., 2016; Hailu et al., 2020). One of the difficulties in measuring HRQoL in this patient group is the lack of specifically developed instruments, that combine assessing the impact of treatment of both TB and HIV, without putting too much emphasis on long-term signs and symptoms in these semi-acutely ill patients. This lack stresses the need for research on the psychometric properties of existing instruments in this population. This is also in line with the increasing importance of including patient-reported outcomes in clinical trials as a valuable way to assess and transparently report on disease-impact in a patient-centered way (Mercieca-Bebber et al., 2018).

A systematic review of reviews by Cooper et al. (2017) indicated that one promising candidate for validly and reliably measuring HRQoL in this population is the Patient Reported Outcomes Quality of Life-HIV (PROQOL-HIV; Cooper et al., 2017). This instrument was developed in 2014 based on a conceptual model emerging from qualitative patient interviews in nine countries including resource-limited settings to overcome the fact that previously used HRQoL instruments were developed before the introduction of ART and focused mainly on resource-rich settings (Duracinsky et al., 2012a). Since the PROQOL-HIV has been shown to be sensitive to sociocultural context, disease stage, and ART, it was selected for the current study. As the current study focuses its attention on patients with HIV-associated TB, we adapted the PROQOL-HIV by adding items related to TB symptoms—resulting in a PROQOL-HIV/TB scale.

As a response to the research needs identified above, we (1) assessed the performance (i.e., factor structure, reliability, validity) of the PROQOL-HIV/TB in measuring HRQoL in patients with HIV-associated TB and subsequently (2) used the PROQOL-HIV/TB to determine the effect of prophylactic prednisone on HRQoL in our patient cohort.

## Materials and methods

### Study context: PredART trial

This study was a sub-study of the PredART trial (Stek et al., 2016; Meintjes et al., 2018), a Phase 3 randomized, double-blind placebo-controlled trial of prophylactic prednisone (40 mg/day for 2 weeks followed by 20 mg/day for 2 weeks, started within 48 h of initiating ART) in patients with HIV-associated TB who were identified being at high risk for paradoxical TB-IRIS (starting ART within 30 days of initiating TB treatment and CD4 cell count ≤100 cells/μl). The primary endpoint was development of paradoxical TB-IRIS within 12 weeks of starting ART as defined by the International Network for the Study of HIV-associated IRIS (INSHI) criteria. Secondary outcomes included mortality and self-reported quality of life. The methods have been published previously and are provided in the protocol and statistical analysis plan (Stek et al., 2016). The trial received ethical approval from the Institutional Review Board of the Institute of Tropical Medicine, Antwerp, Belgium (IRB reference number 882/13), the Ethics Committee of the Antwerp University Hospital, Antwerp, Belgium (B300201317735), and the Faculty of Health Sciences Human Research Ethics Committee of the University of Cape Town, South Africa (UCT HREC reference number 136/2013).

### Sampling and study setting

The trial was conducted at the Khayelitsha Site B HIV-TB clinic and enrolled participants from four different clinics in the area. Khayelitsha is a township of about 400,000 inhabitants, approximately 20 kilometers outside Cape Town, where large numbers of patients start ART annually (i.e., over 40,000 patients have started ART in Khayelitsha since 2002; Kaplan et al., 2017). Inclusion criteria for the trial were (1) being HIV infected, (2) having a CD4 count ≤100 cells/μL, (3) being ART-naïve, (4) having a microbiologic or clinical diagnosis of TB, (5) being on TB treatment for less than 30 days prior to study entry, (6) eligible for ART and consenting to starting ART within 30 days of starting TB treatment and (7) providing written informed consent for screening and again for enrollment into the trial (more detailed information in Appendix 1). A total of 240 adult participants were recruited between August 2013 and February 2016, and randomized to (1) a prednisone arm or (2) a placebo arm. Randomization was performed in a 1:1 ratio with a block size of 8; the randomization sequence was prepared by an independent statistician. Intensive participant follow-up in this study was short (12 weeks) as prednisone was only provided during the first 4 weeks of the trial and the majority of the TB-IRIS cases occur within 3 months. More information on sample size calculation, group allocation and blinding is provided in the published research protocol (Stek et al., 2016).

### Ethics approval

The trial received ethical approval from the Institutional Review Board of the Institute of Tropical Medicine, Antwerp, Belgium (IRB reference number 882/13), the Ethics Committee of the Antwerp University Hospital, Antwerp, Belgium (B300201317735), and the Faculty of Health Sciences Human Research Ethics Committee of the University of Cape Town, South Africa (UCT HREC reference number 136/2013).

### Data

This substudy uses PredART trial data collected at week 0 (start of trial medication and ART), week 4 and week 12 of the trial.

#### Assessment of HRQoL

Firstly, HRQoL was assessed using the validated PROQOL-HIV, with adaptations to reflect TB symptoms—resulting in the PROQOL-HIV/TB scale. This instrument comprised eight dimensions measured by 38 items: (1) physical health & symptoms, (2) bodily changes, (3) social relationships, (4) intimate relationships, (5) stigma, (6) emotional distress, (7) health concerns and (8) treatment impact (Duracinsky et al., 2012b; Sommerland et al., 2017). The scale additionally comprises (1) one item assessing the general health perception (2) and four extra items addressing religious beliefs, finances, having children, and satisfaction with care. These items are not part of the scoring scheme of the 8 sub-scales as defined by Duracinsky et al. (2012b) and are used to gather additional information from the respondent (Duracinsky et al., 2014). We did not use them in our analyses.

Patients rated their HRQoL over the past 2 weeks on a five-point scale ranging from 0 = “never” to 4 = “always.” In order to reliably test HRQoL in our sample, we translated the scale using a systematic procedure: two forward translations from English into isiXhosa, reconciliation, back-translation into English, and final reconciled forward translation (Ravens-Sieberer et al., 2014).

We adapted the scale for use in patients with HIV-associated TB through (1) adding the wording ‘and TB’ to the questions about HIV; (2) adding three TB-specific questions; (3) including symptoms of TB and side effects of TB treatment or prednisone; (4) excluding long-term side effects of ART; and (5) adapting some wording for better comprehension in the local context as a result of the systematic translation procedure. The final PROQOL-HIV/TB scales assessed consisted of 42 items (the 38 items belonging to one of the eight QoL subscales + the three TB-specific items + one item on the general health perception). The adapted PROQOL-HIV/TB instrument (and its scoring scheme) is provided in Appendix 2.

Secondly, to validate the PROQOL-HIV/TB scale one other HRQoL measures was used for comparison: the EQ-5D-3L which consists of (1) EQ-5D descriptive system [Value set of Jelsma et al. (2003) and previously used in South Africa (FlR et al., 2007)]: a standardized instrument covering five generic dimensions of current health (mobility, self-care, usual activities, pain/discomfort, anxiety/depression; Rabin and Charro, 2001; Wouters et al., 2009a,b) and (2) the EQVAS: a visual analogue scale measuring overall health status (Wouters et al., 2007). Given the fact that the EQ-5 descriptive system only includes items measuring physical and emotional aspects of HRQoL, we primarily expect significant correlations with the following subdimensions of the PROQOL-HIV/TB: physical health & symptoms, bodily changes, emotional distress, treatment impact and the general health perception. We expect the EQ-VAS to be especially related to the general health perception.

#### Symptoms

Disease-related symptoms were assessed using the HIV Symptoms Index (HIV-SI; Justice et al., 2001) which measures HIV symptoms for clinical management, patient-oriented research, and adverse drug reporting. The original 20-item instrument was complemented with four items related to TB-IRIS and steroid side effects. Given the physical nature of this Symptoms Index, we primarily expect significant correlations with the following subscales: physical health & symptoms, bodily changes, health concerns, treatment impact and the general health perception.

#### Functional exercise capacity

Functional exercise capacity was assessed in a subset of respondents using the six-minute walk test (6MWT; ATS Committee on Proficiency Standards for Clinical Pulmonary Function Laboratories, 2002) measuring the distance an individual is able to walk over a total of 6 min on a hard, flat surface. Again, we expect the highest correlations with the subscales of the PROQOL-HIV/TB which measure physical aspects of HRQoL.

#### Lung function

Lung function was assessed in a subset of respondents using spirometry, measuring the percentage of the predicted forced expiratory volume in 1 s (FEV1%; Miller et al., 2005). The lung function results are expected to correlate mores strongly with the physical aspects of HRQoL.

#### Other relevant variables

These included sex and age of the respondents, baseline CD4 cell count and HIV viral load, and randomization arm.

### Data analysis

#### Testing the PROQOL-HIV/TB

Internal construct validity was assessed by a series of separate Confirmatory Factor Analyses (CFAs) (Muthén and Muthén, 1998–2007). For each of the eight QoL subscales (Duracinsky et al., 2012b) and for each of the three time points, items that did not successfully load [standardized factor loadings (λ) > 0.40] onto the theoretical QoL (sub)domain were removed (Brown, 2006). We used the following fit indices: the Tucker Lewis Index (TLI), the comparative fit index (CFI), the Root Mean Square Error of Approximation (RMSEA), and the Standardized Root Mean Square Residual (SRMR). Following the recommendations of Hu and Bentler (1999), two of the following three criteria had to be met for satisfactory global model fit to be attained: CFI/TLI ≥ 0.95, RMSEA ≤0.06, and SRMR ≤0.08 (Hu and Bentler, 1999; Shi et al., 2021).

Reliability of the different HRQoL scales was measured by Cronbach’s Alpha coefficients. For a scale to be considered consistent, the value of the coefficient has to be above 0.70 (Bland, 1997). Cronbach’s alpha has been criticized as a measure of reliability as the index is not derived from parameters of a factor model but uses variances and covariances among the items (Sijtsma, 2008; Raykov, 2012). It is valid when the items are unidimensional and at least tau-equivalent, and with uncorrelated residuals, but is otherwise problematic. Therefore, we also report the factor determinacy score coefficient (>0.8) and provide the correlation between the estimated and true factor scores to evaluate how well the factor is measured (Tabachnick and Fidell, 2013).

We tested whether the eight PROQOL-HIV subscales had the same meaning at each of the three time points through testing for factorial invariance: (1) configural, (2) metric and (3) scalar invariance of the targeted construct across the three time points. Configural invariance requires association of the PROQOL-HIV factors with the same items across the three time points. Metric invariance requires the corresponding factor loadings to be equal across time points, testing whether the common factors have the same meaning across time. Scalar invariance requires equal item intercepts across time. The lack of scalar invariance signals the presence of item bias (or differential item functioning). Its presence states that comparing factor means across time points is possible (Gregorich, 2006; Dimitrov, 2010). The restrictions for equality of specific parameters across time are imposed one after the other, thus producing nested models that are tested against each other using chi-square difference testing (Muthen and Muthen, 2005).

External construct validity was tested by assessing the relationship between the different HRQoL subscales and relevant correlates—namely (1) the EQ-5D descriptive system, (2) the EQ-VAS, (3) the HIV-SI, and the results from (4) the 6MWT and (5) the lung function test (Muthén and Muthén, 1998–2007). We expect the PROQOL-HIV subscales to be significantly and positively correlated with the other HRQoL scales, the 6MWT and the lung function test, and negatively correlated with the Symptoms Index.

#### Mixed model

To assess whether prednisone significantly affected the eight different HRQoL-dimensions using the PROQOL-HIV/TB, 16 separate mixed models were fitted, two for each dimension (applying both intention-to-treat (ITT) and all-patients-treated approaches). Each model included a fixed effect for time, arm and their interaction (time*arm) and a random intercept per participant. A significant value of *p* (<0.05) for the interaction indicated a significantly different evolution over time for the two arms.

## Results

### Participant characteristics and presentation of items

The characteristics of participants are shown in Table 1. Table 2 displays the different items ascribed to the respective HRQoL subscales and the spread of participants’ responses at the different time points.

**Table 1:** Baseline characteristics (*N* = 240).

	**Prednisone arm (*n* = 120)**	**Placebo arm (*n* = 120)**
Gender		
Male (%)	71 (59.2)	73 (60.8)
Female (%)	49 (40.8)	47 (39.2)
Median age (years) (IQR)	36 (31–42)	36 (29–42)
Median baseline CD4 cell count (cells/μl) (IQR)	51 (27–84)	49 (23–88)
Median baseline HIV viral load (log_10_ copies/ml) (IQR)	5.5 (5.2–5.9)	5.6 (5.2–5.9)

**Table 2:** Confirmatory factor analyses: eight PROQOL-HIV/TB subscales, factor loadings, reliability estimate and goodness-of-fit indices.

**Physical health & symptoms**	**Standardized loading** ^†^ **Week 0**	**Means** ^‡^ Alpha: 0.810^§^	**Standardized loading Week 4**	**Means** Alpha: 0.808	**Standardized loading Week 12**	**Means** Alpha: 0.853
Item 2	0.533	1.523	0.626	1.125	0.626	0.689
Item 3	0.557	1.068	0.530	1.183	0.616	0.757
Item 4	0.527	0.614	0.519	0.535	0.639	0.454
Item 5	0.474	0.847	0.466	0.843	0.612	0.710
Item 6	0.707	1.166	0.634	1.005	0.785	0.713
Item 7	0.706	1.759	0.685	1.423	0.720	0.981
Item 8	0.571	0.991	0.484	0.981	0.600	0.718
Item 9	0.539	1.098	0.615	1.084	0.639	0.772
Item 10	0.533	1.519	0.522	1.199	0.554	0.854
**Bodily changes**		Alpha: 0.523		Alpha: 0.605		Alpha: 0.631
Item 11	0.639	1.560	0.542	1.070	0.640	0.617
Item 12	0.398	1.026	0.505	0.934	0.405	1.044
Item 13	0.421	0.457	0.421	0.400	0.629	0.459
Item 14	0.592	1.474	0.653	1.094	0.655	0.734
**Social relationships**		Alpha: 0.665		Alpha: 0.719		Alpha: 0.760
Item 15	0.680	0.701	0.656	0.587	0.728	0.362
Item 16	0.731	0.654	0.859	0.488	0.853	0.360
**Intimate relationships**		Alpha: 0.673		Alpha: 0.805		Alpha: 0.777
Item 17	0.481	0.735	0.668	0.723	0.700	0.625
Item 18	0.727	1.615	0.832	1.160	0.784	1.072
Item 19	0.731	1.013	0.797	0.991	0.743	0.769
**Stigma**		Alpha: 0.625		Alpha: 0.661		Alpha: 0.714
Item 20	0.605	1.000	0.487	0.808	0.728	0.628
Item 21	0.520	0.421	0.536	0.420	0.707	0.394
Item 22	0.513	1.813	0.602	1.608	0.545	1.529
Item 23	0.605		0.697	0.882	0.661	0.821
**Emotional distress**		Alpha: 0.733		Alpha: 0.787		Alpha: 0.786
Item 24	0.781	1.229	0.751	1.085	0.764	0.783
Item 25	0.762	1.134	0.878	1.052	0.856	0.888
Item 26	0.464	0.782	0.527	0.842	0.583	0.634
Item 27	0.546	0.788	0.651	0.701	0.592	0.490
**Health concern**		Alpha: 0.803		Alpha: 0.789		Alpha: 0.815
Item 28	0.605	0.952	0.458	0.784	0.488	0.769
Item 29	0.465	0.863	0.435	0.796	0.482	0.773
Item 30	0.689	1.211	0.754	1.120	0.805	1.029
Item 31	0.690	1.621	0.812	1.624	0.764	1.476
Item 32	0.596	1.927	0.674	1.865	0.694	1.657
**Treatment impact**		Alpha: 0.796		Alpha: 0.796		Alpha: 0.797
Item 37	0.728	0.613	0.507	0.533	0.533	0.449
Item 38						
Item 39	0.521	0.517	0.605	0.664	0.493	0.560
Item 40	0.539	0.817	0.642	0.873	0.553	0.755
Item 41	0.571	0.748	0.678	0.844	0.541	0.691
Item 42	0.676	0.413	0.572	0.559	0.667	0.424
Item 43						
Item 44	0.582	0.155	0.492	0.329	0.542	0.229
Item 45	0.425	0.368	0.572	0.500	0.654	0.371
Item 46	0.438	0.180	0.356	0.225	0.375	0.278
	**SRMR** ^*^	**RMSEA** ^*^	**CFI** ^*^	**TLI** ^*^		
**Week 0**	0.068	0.050	0.842	0.824		
**Week 4**	0.074	0.054	0.837	0.819		
**Week 12**	0.072	0.060	0.820	0.800		

† The standardized factor loadings can be interpreted as the correlation between the items (of the PROQOL-HIV/TB scale) and the latent factor (the different subdimensions).

‡ These are the mean scores on the individual PROQOL-HIV/TB items.

§ The Cronbach’s alpha (tau-equivalent reliability) is an estimate of the reliability (internal consistency) of each subscale.

* Standardized Root Mean Square Residual (SRMR), Root Mean Square Error of Approximation (RMSEA), Tucker Lewis Index (TLI), and the Comparative Fit Index (CFI).

### Confirmatory factor analyses: Internal construct validity confirmed

Table 2 shows the factor loadings of the different items onto the different theory-based scales. At week 0, the original 8 subscale model did not fit the data well. Item 38 (‘I have been satisfied with my medicine’) did not load well onto the Treatment Impact factor (λ = 0.264), probably because it is the only item of the scale that is reversely coded. Item 43 (‘I have had to hide in order to take my medicine’) also did not load well onto this subscale. Both items were deleted from the Treatment Impact factor. We allowed for error covariances between items that were similarly worded: items 11, 12 and 13 (all starting with “I have been bothered by …”); items 28 and 29 (“… was on my mind”); items 31 and 32 (“I have been afraid…”); and items 40, 41 and 42 (all mentioning treatment aspects potentially perceived as “bothering” the respondent). We repeated this procedure for the factor models at week 4 and week 12 with the same factor structure as the outcomes. Table 2 shows that these adapted factor models displayed an acceptable fit to the data.

### Scale reliability confirmed

The resulting factors were subjected to a reliability analysis (Table 2). Overall, reliability was good. However, at week 0, four subscales displayed questionable reliability levels with Cronbach’s alpha coefficient < 0.7. At week 4, only two subscales failed to meet this criterion and at week 12 only the Bodily Changes subscale failed the test. The relative low alpha levels are likely related to the low number of items in these subscales. The Physical Health & Symptoms, Emotional Distress, Health Concern and Treatment Impact subscales displayed good reliability at all-time points. All factor determinacy scores were above 0.8, indicating that the observed variables account for substantial variance in the factor scores.

### Factorial invariance: Metric invariance confirmed

Subsequently, the factorial invariance of the different QoL subscales was tested across time. First, configural invariance of the factor structure across the three time points was established, supported by the satisfactory model fit of the three above-tested models. Next, metric invariance was tested, requiring corresponding factor loadings to be equal across groups, using a chi-square difference (Δχ^2^) test for each pair of two nested models. Metric invariance of the eight HRQoL subscales was tested for each subscale separately. The small sample size (*n* = 240 at week 0) did not allow for simultaneous testing in one pair of nested models. None of the eight tested subscales displayed a significant increase in χ^2^, indicating invariance of the factor loadings across the three time points. This provides evidence that corresponding common factors have the same meaning across time. In a final step, scalar invariance was tested requiring equal factor loadings and equal indicator intercepts (i.e., indicator means) across time. This additional restriction did result in a significant increase in χ^2^ for all other QoL subscales except for the Health Concern subscale. This means that only the Health Concern subscale was fully scalar invariant over time, i.e., it was the only sub-scale allowing for comparisons of its latent means across the three time points.

#### Score construction

Because of this insufficient scalar invariance over time and to maximize comparability with previous studies employing the PROQOL-HIV, we constructed the scores as suggested by its developers (Duracinsky et al., 2012a,b): summary scores were calculated for each dimension (HRQoL subscale) and a global score for the questionnaire as a whole. Scores were computed as the sum of corresponding items scores and were standardized on a 100-point scale (0 = “worst” to 100 = “best” HIV HRQoL; see Table 3).

**Table 3:** Mean PROQOL-HIV/TB sub-scores in the prednisone and placebo arm.

	**Prednisone arm**			**Placebo arm**		
**Mean**	**Week 0**	**Week 4**	**Week 12**	**Week 0**	**Week 4**	**Week 12**
Physical health & symptoms	68.0	75.7	80.3	72.0	71.5	83.6
Bodily changes	68.8	77.5	81.9	74.6	78.7	82.5
Social relationships	79.9	83.9	91.1	86.0	89.4	90.8
Intimate relationships	71.4	72.1	76.9	72.5	80.3	81.9
Stigma	71.6	75.9	78.6	75.6	77.5	79.7
Emotional Distress	73.5	78.2	83.1	78.4	77.3	82.6
Health concerns	65.1	69.6	70.4	69.5	69.8	72.6
Treatment impact^†^	86.4	84.4	86.7	89.6	87.3	89.6
Global score	73.2	78.0	80.8	77.4	78.2	83.6

† The treatment impact subscale in the adapted PROQOL-HIV/TB scale refers to both HIV and TB treatment. All included patients had started TB treatment between 2 and 4 weeks prior to the start of the study.

### External construct validity confirmed

The individual coefficients of correlation between the eight HRQoL subscales, the global score and the other relevant correlates are listed in Table 4. Overall, the high proportion of (expected) significant coefficients supports the external validity of our subscales, indicating that they are indeed measuring what they intend to measure: differing dimensions of the health-related quality of life of patients with HIV-associated TB.

**Table 4:** Correlations between the HRQOL subscales and the global score and the relevant correlates: (1) EQ-5D descriptive system (d.s.), (2) EQ-VAS, (3) the HIV-SI, and the results from (4) the lung function test (FEV 1%) and (5) the 6 min walking test (6MWT).

	PROQOL-HIV/TB								
**Week 0**	**PHS**	**BC**	**SR**	**IR**	**ST**	**ED**	**HC**	**TI**	**GS**
EQ-VAS	0.297^***^	0.181^**^	0.181^**^	0.059	0.136^*^	0.147^*^	0.060	0.061	0.201^**^
EQ-5D d.s.	0.496^***^	0.358^***^	0.273^***^	0.244^***^	0.250^***^	0.241^***^	0.244^***^	0.267^***^	0.456^***^
HIV-SI	−0.540^***^	−0.439^***^	−0.326^***^	−0.341^***^	−0.325^***^	−0.442^***^	−0.246^**^	−0.305^***^	−0.562^***^
FEV1%	0.067	−0.066	−0.137	−0.023	0.035	0.002	0.052	−0.243^*^	−0.067
6MWT	0.330^**^	0.152	0.161	−0.089	0.137	0.113	0.065	0.025	0.156
**Week 4**	**PHS**	**BC**	**SR**	**IR**	**ST**	**ED**	**HC**	**TI**	**GS**
EQ-VAS	0.432^***^	0.221^**^	0.265^***^	0.192^**^	0.026	0.147^*^	0.078	0.162^*^	0.311^***^
EQ-5D d.s.	0.490^***^	0.335^***^	0.327^***^	0.380^***^	0.144^*^	0.298^***^	0.230^*^	0.321^***^	0.472^***^
HIV-SI	−0.714^***^	−0.650^***^	−0.386^***^	−0.460^***^	−0.391^***^	−0.553^***^	−0.429^***^	−0.596^***^	−0.770^***^
FEV1%	0.170	−0.044	0.043	0.028	0.046	−0.101	−0.155	−0.078	−0.114
6MWT	0.364^*^ ^*^	0.089	0.249^*^	0.038	0.110	0.077	0.137	−0.043	0.168
**Week 12**	**PHS**	**BC**	**SR**	**IR**	**ST**	**ED**	**HC**	**TI**	**GS**
EQ-VAS	0.338^***^	0.164^*^	0.119	0.200^**^	0.061	0.201^**^	0.159^*^	0.119	0.277^***^
EQ-5D d.s.	0.546^***^	0.390^***^	0.292^***^	0.288^***^	0.197^**^	0.384^***^	0.301^***^	0.345^***^	0.495^***^
HIV-SI	−0.711^***^	−0.643^***^	−0.430^***^	−0.469^***^	−0.310^***^	−0.469^***^	−0.448^***^	−0.512^***^	−0.687^***^
FEV1%	0.008	−0.153	−0.100	0.156	−0.066	−0.116	−0.066	−0.050	−0.058
6MWT	0.206^*^	0.108	0.046	0.199^*^	−0.045	0.012	0.006	0.070	0.098

* *p* < 0.05.

** *p* < 0.01.

*** *p* < 0.001.

PHS, physical health and symptoms; BC, bodily changes; SR, social relationships; IR, intimate relationships; ED, emotional distress; HC, health concern; TI, treatment impact; GS, global score.

### Mixed models: Significant impact on physical health and symptoms subscale

Table 3 shows the mean PROQOL-HIV/TB sub-scores at the three time points in each study arm revealing steady increases in almost all QoL-subdimensions across both groups. This is a consequence of the patients being on the correct treatments (anti tubercular and antiretroviral) for their underlying condition. Table 5 shows the impact of prednisone on HRQoL (i.e., the outcomes of the mixed effects models with fixed effects for time, intervention and their interaction and a random intercept). The results display very little differences between the prednisone and placebo arms. The prednisone arm is statistically significantly associated only with changes in ‘Physical Health and Symptoms’ (PHS) both in intent-to-treat and in all-patients-treated analysis.

**Table 5:** Impact of prednisone on different dimensions of quality of life—ITT (*n* = 240) and all-patients-treated (*n* = 238) analyses.

		**Intent-to-treat**			**All patients**		
		**Estimate**	**95% C.I.**	***p*-value**	**Estimate**	**95% C.I.**	***p*-value**
**Physical Health and Symptoms**							
Baseline difference^†^		−3.90	(−8.97, 1.17)	0.13	−4.33	(−9.39, 0.73)	0.093
Week in Placebo^‡^				<0.001			<0.001
	Week 4	−1.00	(−4.84, 2.84)		−1.33	(−5.18, 2.52)	
	Week 12	10.81	(7.01, 14.61)		10.50	(6.69, 14.30)	
Week: Prednisone *vs*. Placebo^§^				0.009			0.007
	Week 4	7.92	(2.57, 13.28)		8.25	(2.90, 13.60)	
	Week 12	1.18	(−4.19, 6.56)		1.50	(−3.88, 6.87)	
**Body changes**							
Baseline difference		−5.89	(−11.13, −0.65)	0.028	−6.32	(−11.56, −1.07)	0.018
Week in Placebo				0.003			0.003
	Week 4	4.28	(−0.08, 8.63)		4.21	(−0.18, 8.60)	
	Week 12	7.41	(3.14, 11.68)		7.44	(3.14, 11.74)	
Week: Prednisone *vs*. Placebo				0.14			0.14
	Week 4	4.21	(−1.87, 10.29)		4.34	(−1.77, 10.45)	
	Week 12	5.95	(−0.17, 12.07)		5.98	(−0.17, 12.14)	
**Social relationships**							
Baseline difference		−6.02	(−11.65, −0.38)	0.036	−6.71	(−12.32, −1.10)	0.019
Week in Placebo				0.17			0.31
	Week 4	3.02	(−1.85, 7.88)		2.37	(−2.43, 7.18)	
	Week 12	4.48	(−0.33, 9.28)		3.62	(−1.13, 8.36)	
Week: Prednisone *vs*. Placebo				0.17			0.11
	Week 4	0.50	(−6.32, 7.33)		1.10	(−5.64, 7.84)	
	Week 12	6.00	(−0.89, 12.89)		6.81	(0.01, 13.62)	
**Intimate relationships**							
Baseline difference		−1.53	(−8.37, 5.30)	0.66	−1.77	(−8.65, 5.11)	0.61
Week in Placebo				0.007			0.012
	Week 4	6.71	(1.07, 12.34)		6.37	(0.71, 12.03)	
	Week 12	8.45	(2.87, 14.02)		8.05	(2.45, 13.65)	
Week: Prednisone *vs*. Placebo				0.33			0.38
	Week 4	−5.94	(−13.82, 1.94)		−5.59	(−13.49, 2.32)	
	Week 12	−2.61	(−10.56, 5.34)		−2.20	(−10.17, 5.77)	
**Stigma**							
Baseline difference		−3.93	(−9.61, 1.75)	0.17	−4.09	(−9.80, 1.63)	0.16
Week in Placebo				0.27			0.33
	Week 4	2.05	(−2.59, 6.69)		1.76	(−2.91, 6.43)	
	Week 12	3.75	(−0.82, 8.32)		3.48	(−1.12, 8.08)	
Week: Prednisone *vs*. Placebo				0.53			0.47
	Week 4	2.49	(−3.95, 8.93)		2.83	(−3.63, 9.30)	
	Week 12	3.62	(−2.86, 10.10)		3.94	(−2.57, 10.44)	
**Emotional distress**							
Baseline difference		−5.06	(−10.65, 0.54)	0.077	−5.33	(−10.95, 0.29)	0.063
Week in Placebo				0.056			0.064
	Week 4	−1.01	(−5.55, 3.52)		−1.58	(−6.11, 2.95)	
	Week 12	4.27	(−0.21, 8.74)		3.77	(−0.70, 8.23)	
Week: Prednisone *vs*. Placebo				0.12			0.081
	Week 4	6.01	(−0.36, 12.39)		6.56	(0.20, 12.91)	
	Week 12	5.39	(−1.01, 11.78)		5.86	(−0.51, 12.23)	
**Health concerns**							
Baseline difference		−4.69	(−10.92, 1.54)	0.14	−4.86	(−11.12, 1.40)	0.13
Week in Placebo				0.35			0.38
	Week 4	−0.02	(−4.82, 4.78)		−0.11	(−4.94, 4.73)	
	Week 12	3.07	(−1.69, 7.82)		2.91	(−1.88, 7.69)	
Week: Prednisone *vs*. Placebo				0.37			0.36
	Week 4	5.00	(−1.93, 11.93)		5.09	(−1.87, 12.05)	
	Week 12	2.50	(−4.38, 9.38)		2.65	(−4.25, 9.56)	
**Treatment impact**							
Baseline difference		−3.02	(−6.85, 0.81)	0.12	−2.93	(−6.79, 0.92)	0.13
Week in Placebo				0.19			0.20
	Week 4	−2.52	(−5.25, 0.23)		−2.45	(−5.20, 0.30)	
	Week 12	−0.91	(−3.68, 1.85)		−0.71	(−3.49, 2.07)	
Week: Prednisone *vs*. Placebo				0.79			0.85
	Week 4	0.21	(−3.70, 4.12)		0.12	(−3.80, 4.05)	
	Week 12	1.30	(−2.65, 5.25)		1.07	(−2.89, 5.03)	
**General health perception**							
Baseline difference		−4.53	(−8.64, −0.43)	0.031	−4.84	(−8.95, −0.73)	0.021
Week in Placebo				<0.001			<0.001
	Week 4	0.86	(−2.14, 3.87)		0.56	(−2.45, 3.58)	
	Week 12	5.89	(2.92, 8.87)		5.62	(2.63, 8.60)	
Week: Prednisone *vs*. Placebo				0.17			0.13
	Week 4	4.09	(−0.18, 8.36)		4.39	(0.12, 8.66)	
	Week 12	2.11	(−2.11, 6.33)		2.38	(−1.84, 6.61)	

† Difference in [HRQoL subdomain] at week 0 for participants receiving prednisone compared to those receiving placebo.

‡ Mean change in [HRQoL subdomain] from week 0 in participants in placebo arm.

§ Difference in mean change in [HRQoL subdomain] from week 0 for participants in prednisone arm compared to those in placebo arm.

**Figure 1:**
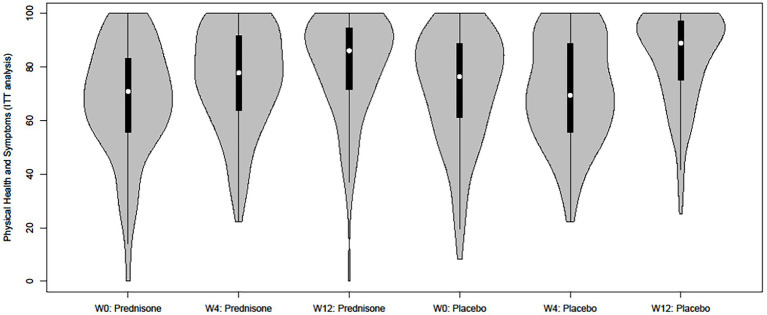
Grouped violin plot of the Physical Health and Symptoms score per intervention arm and time point (intent-to-treat).

**Figure 2:**
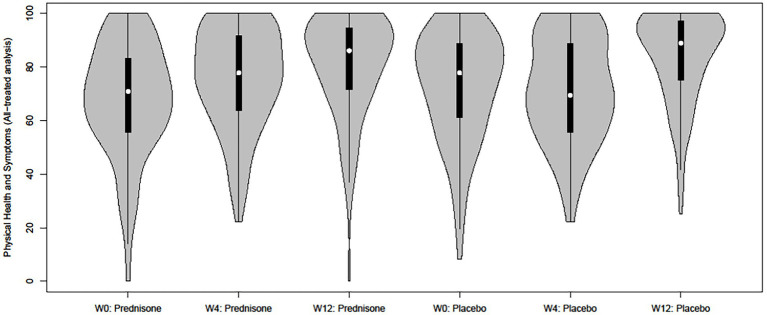
Grouped violin plot of the Physical Health and Symptoms score per intervention arm and time point (all patients treated).

A detailed analysis of the difference in PHS between arms reveals a different time pattern in the control versus the intervention group: there is no significant change in PHS score over time from week 0 to week 4 in the placebo arm [mean difference of −1.00 (−4.84, 2.84)]. The PHS score change between weeks 0 and 4 significantly differs between the prednisone and placebo arms (a difference of 7.92 and a significant interaction term) indicating that prednisone stimulates the PHS subdomain mainly in these first 4 weeks that participants were on the intervention. This significant difference between the two groups disappears by week 12, indicating that the increase in this HRQoL subdomain seems to occur earlier in the intervention group compared to the control group. These trends are also visible in the violin plot in Figure 1 (intent-to-treat) and Figure 2 (all-patients).

## Discussion

We amended the PROQOL-HIV, an HIV-specific HRQoL instrument, to include features related to TB and to be better suited for low-and middle-income countries: the PROQOL-HIV/TB. We employed data from a randomized, double-blind, placebo-controlled trial (NCT01924286, registered at clinicaltrials.gov) conducted in South Africa to evaluate the performance of the PROQOL-HIV/TB: we assessed whether the positive impact of prophylactic prednisone in preventing TB-IRIS translated into improvements in patients’ self-reported HRQOL.

Recently, and within the framework of the ongoing debate on assessing the target of the 4th 90 as part of the HIV continuum of care, an international consensus panel identified the need for health systems to pay careful attention to language, culture, and local and individual priorities regarding well-being when assessing HRQoL outcomes (Lazarus et al., 2021). Our findings contribute to achieving this goal in patients co-infected with HIV and TB in resource-limited settings. We have shown that the PROQOL-HIV/TB adequately measures different aspects of self-reported HRQoL in patients with HIV-associated TB, as demonstrated by the internal construct validity, the internal reliability and the external validity of the eight subscales. This is in line with a recent systematic review showing that the relatively new PROQOL-HIV is cross-culturally one of the most valid measures with great relevance for people living with HIV since items were developed with extensive input from people living with HIV across nine countries (Cooper et al., 2017). With the PROQOL-HIV/TB adapted for this specific study and setting, we have provided an instrument to measure HRQoL in patients with HIV-associated TB which is applicable in low-middle-income countries, where the burden of HIV and TB is greatest.

Previous studies have indicated that self-reported improvements in HRQoL (or the lack thereof) are important predictors of ART adherence (Mannheimer et al., 2005; Holmes et al., 2007). It has also been demonstrated that high adherence levels during the initial weeks of ART are predictive of not missing clinical visits (Jiamsakul et al., 2018) and durable virologic suppression (Flynn et al., 2007). Prednisone used as prophylaxis to prevent TB-IRIS could play a role in improving HRQoL– as measured by the amended PROQOL-HIV/TB – but only in the sub-dimension ‘physical health & symptoms’. Patients’ self-perceived physical health status was boosted in the intervention group during the first 4 weeks of ART, while patients in the control group lagged behind and caught up after 12 weeks of ART. This could potentially be linked to the fact that, as reported in the paper on the main trial results (Meintjes et al., 2018), fewer people in the Prednisone-arm had to interrupt ART and/or TB treatment due to side-effects (doctor-initiated interruption) compared to the control-arm. Alternatively, short-term corticosteroid use can improve non-specific well-being and performance during submaximal exercise (Arlettaz et al., 2007), which may result in better physical health and symptoms.

We observed no statistically significant differences between the prednisone and the placebo arms for any of the other HRQoL subscales. Prednisone most likely exerts its positive effect on physical HRQoL by preventing TB-IRIS. Prevention of TB-IRIS has clear physical benefits which in the short period of time may not directly translate into perceived improvements in psychosocial aspects of HRQoL. ‘Stigma’, ‘social relationships’ or ‘emotional distress’ can be considered distal constructs requiring structural or psychosocial changes to improve over time, which are not directly attributable to medical interventions (Rzeszutek and Gruszczyńska, 2018).

### Limitations

The study is subject to an important limitation: the trial’s power calculations were aligned to the primary endpoint of the trial, namely the development of TB-IRIS. The trial may thus not be sufficiently powered to detect differences in all dimensions of HRQoL. In addition, our findings may not be generalizable to inpatients—however, most patients with HIV-associated TB are ambulatory. This limited sample size also impacted on the validation of the instrument and its subscales. Another limitation is the limited geographic (and therefore possibly socio-demographic) extent of the study—potentially limiting the generalizability of the study findings. In addition, a comprehensive validation of the instrument would have also included a qualitative component. Ideally we would also have separated the development of the adapted PROQOL-HIV/TB from the testing of the instrument in two substudies employing different datasets. The substudy may be further limited by the fact that study participants who were diagnosed with TB-IRIS during follow-up received open-label prednisone as treatment. This may have reduced the effect of prophylactic prednisone over time, potentially explaining why a significant change was observed at week 4 but no longer at week 12.

## Conclusion

To our knowledge, this is the first study to (1) explicitly test an adapted HRQoL scale in patients with HIV-associated TB at high risk of TB-IRIS and (2) assess the impact of prednisone on HRQoL in this population—resulting in high methodological and practical relevance. Methodologically, the study amended the original instrument and produced a valid and reliable instrument to measure different dimensions of HRQoL in patients with HIV-associated TB—the PROQOL-HIV/TB. Practically, we demonstrated its applicability by using the newly developed instrument to assess the impact of prednisone on different dimensions of HRQoL in a sample of patients being treated for TB and starting ART at high risk of developing TB-IRIS.

## Data availability statement

The raw data supporting the conclusions of this article will be made available by the authors, without undue reservation.

## Ethics statement

The studies involving human participants were reviewed and approved by The trial received ethical approval from the Institutional Review Board of the Institute of Tropical Medicine, Antwerp, Belgium (IRB reference number 882/13), the Ethics Committee of the Antwerp University Hospital, Antwerp, Belgium (B300201317735), and the Faculty of Health Sciences Human Research Ethics Committee of the University of Cape Town, South Africa (UCT HREC reference number 136/2013). The patients/participants provided their written informed consent to participate in this study.

## Author contributions

All authors contributed to conception and design of the study. CN, LL, CS, and EW contributed to the scale adaptation. JB and EW performed the analyses. EW and CS wrote the first draft of the manuscript. All authors contributed to article and approved the submitted version.

## Funding

This work was supported by the European and Developing Countries Clinical Trials Partnership through a Strategic Primer Grant (SP.2011.41304.074) that was awarded to the University of Cape Town, the Institute of Tropical Medicine, and Imperial College London, funding from the Department of Science and Technology of the government of South Africa, grants (098316, 084323, 104803, 203135) from the Wellcome Trust, and a doctoral fellowship (awarded to Stek) from the Institute for Tropical Medicine. Meintjes is supported by a grant (64787) from the South African Research Chairs Initiative of the Department of Science and Technology and the National Research Foundation (NRF) of South Africa, NRF incentive funding (UID: 85858), and the South African Medical Research Council through its TB and HIV Collaborating Centres Programme, with a grant (RFA# SAMRC-RFA-CC: TB/HIV/AIDS-01-2014) funded by the National Department of Health. Wilkinson is supported by a grant (10218) from the Francis Crick Institute, which is funded by Wellcome Trust, Research Councils UK, and Cancer Research UK.

## Conflict of interest

The authors declare that the research was conducted in the absence of any commercial or financial relationships that could be construed as a potential conflict of interest.

## Publisher’s note

All claims expressed in this article are solely those of the authors and do not necessarily represent those of their affiliated organizations, or those of the publisher, the editors and the reviewers. Any product that may be evaluated in this article, or claim that may be made by its manufacturer, is not guaranteed or endorsed by the publisher.
